# Exposure–response analysis and simulation of lenvatinib safety and efficacy in patients with radioiodine-refractory differentiated thyroid cancer

**DOI:** 10.1007/s00280-018-3687-4

**Published:** 2018-09-22

**Authors:** Seiichi Hayato, Robert Shumaker, Jim Ferry, Terri Binder, Corina E. Dutcus, Ziad Hussein

**Affiliations:** 1 Eisai Co., Ltd., Koishikawa 4-6-10, Bunkyo-ku, Tokyo, 112-8088 Japan; 2 Eisai Inc., Woodcliff Lake, NJ USA; 3 Eisai Ltd., Hatfield, UK

**Keywords:** Thyroid cancer, Radioiodine-refractory; lenvatinib, Exposure–response modeling

## Abstract

**Purpose:**

Once-daily lenvatinib 24 mg is the approved dose for radioiodine-refractory differentiated thyroid cancer. In a phase 3 trial with lenvatinib, the starting dose of 24 mg was associated with a relatively high incidence of adverse events that required dose reductions. We used an exposure–response model to investigate the risk–benefit of different dosing regimens for lenvatinib.

**Methods:**

A population pharmacokinetics/pharmacodynamics modeling analysis was used to simulate the potential benefit of lower starting doses to retain efficacy with improved safety. The seven lenvatinib regimens tested were: 24 mg; and 20 mg, 18 mg, and 14 mg, all with or without up-titration to 24 mg. Exposure–response models for efficacy and safety were created using a 24-week time course.

**Results:**

The approved dose of lenvatinib at 24 mg, predicted the best efficacy. However, the lenvatinib dosing regimens of 14 mg with up-titration or 18 mg without up-titration potentially provides comparable efficacy (objective response rate at 24 weeks) and a better safety profile.

**Conclusions:**

Treatment with lenvatinib at starting doses lower than the approved once-daily 24 mg dose could provide comparable antitumor efficacy and a similar or better safety profile. Based on the results from this modeling and simulation study, a comparator dose of lenvatinib 18 mg without up-titration was selected for evaluation in a clinical trial.

**Electronic supplementary material:**

The online version of this article (10.1007/s00280-018-3687-4) contains supplementary material, which is available to authorized users.

## Introduction

Of the various thyroid cancers, advanced differentiated thyroid cancer (DTC) represents a small, but difficult-to-treat patient population, particularly when the disease becomes radioiodine refractory (RR) [1]. The development of therapeutic agents targeting signaling pathways that are associated with the development and progression of thyroid cancer has offered a new strategy to these patients [1].

Lenvatinib is an oral, multitargeted, tyrosine kinase inhibitor of the vascular endothelial growth factor receptor (VEGFR) 1, 2, and 3; fibroblast growth factor receptor (FGFR) 1–4; platelet-derived growth factor receptor (PDGFR)-α; and the RET and KIT signaling pathways [2–4]. In the phase 3 Study of (E7080) Lenvatinib in Differentiated Cancer of the Thyroid (SELECT) that enrolled patients with RR-DTC, lenvatinib, at a maximum starting dose of 24 mg once daily, significantly prolonged progression-free survival (PFS) vs. placebo (median 18.3 vs. 3.6 months, respectively; hazard ratio 0.21; 99% confidence interval, 0.14–0.31; *P* < 0.001) and was associated with a significantly better response rate (64.8% vs. 1.5%, respectively) [5].

Lenvatinib is approved for the treatment of RR-DTC in more than 50 countries, including the United States, European Union, Japan, Australia, Switzerland, and South Korea [6, 7]. Moreover, lenvatinib is the preferred treatment over sorafenib for progressive and/or symptomatic RR-DTC [8]. The key phase 2 and phase 3 trials of lenvatinib in patients with RR-DTC used the maximum tolerated dose of 24 mg once daily [5, 9], which is, therefore, the recommended daily dose [10]. However, this dose was associated with a relatively high incidence of adverse events (AEs) requiring either supplemental targeted therapy, dose interruption, and/or dose reduction [5, 9]. Most AEs were manageable with AE-targeted therapy or dose reduction [5]; however, the dose for maximum efficacy of lenvatinib must be balanced against its safety profile [11].

Both the United States Food and Drug Administration (FDA) and the European Medicines Agency (EMA) requested a postmarketing study in patients with RR-DTC to evaluate the risk–benefit of initiating lenvatinib treatment using lower starting doses of lenvatinib (14 and 20 mg without up-titration) compared to the approved 24 mg dose level, using objective response rate (ORR) at week 24 as the primary efficacy end point (Study 211; NCT02702388) [12]. At the time of protocol design for this postmarketing requirement study, the lenvatinib 14 and 20 mg starting doses were proposed by the sponsor and agreed to by the 2 regulatory agencies based mainly on pharmacokinetic simulations for exposure differences. This trial was initiated and consisted of three treatment arms (14, 20 and 24 mg) but was terminated in August 2016 and restarted with only two doses of 18 and 24 mg without up-titration, which was agreed to by the regulatory agencies.

Exposure–response models can be useful to investigate drug effects on tumor response and AEs, and provide an initial framework for investigating the risk–benefit of different dosing strategies [13]. Here, we report an exposure–response modeling and simulation study using clinical trial data from SELECT, a phase 3 trial of lenvatinib in patients with RR-DTC [5] and results of a previous population pharmacokinetic (PK) model [7]. The aim of the study was to describe exposure–response models for the time course of tumor size and for dose-altering AEs in patients with RR-DTC, and to simulate the potential clinical benefit of lower starting doses of lenvatinib, with and without up-titration, including the 14 and 20 mg starting doses agreed to by the FDA and EMA for the postmarketing requirement study.

## Methods

### Data and population PK model

The population PK model used pooled data from 15 phase 1, 2, and 3 clinical studies that enrolled both healthy volunteers and patients with solid tumors, thyroid cancer, or RR-DTC [7]. The patient characteristics, data set, and final population PK model are described in detail in this previous publication [7]. As noted in Gupta et al. [7], all patients had provided written informed consent. All studies were conducted in accordance with the International Conference on Harmonization Good Clinical Practice guidelines and were approved by the appropriate independent review boards. In the current study, this population PK model was used to derive individual PK parameters and lenvatinib exposure in patients from SELECT, which were subsequently used in the exposure–response analysis datasets.

### Exposure–response model for efficacy: time course of tumor size

A total of 2373 tumor assessment reports by independent radiologic review were included from patients with RR-DTC from the pivotal SELECT study for whom baseline and at least 1 postdose tumor assessment were available. Of the 392 patients that participated in SELECT, 17 patients did not have any postdose tumor assessments available. Furthermore, 1 patient did not have PK data. Consequently, the final dataset included 374 patients.

Of the 374 patients, 248 received lenvatinib and 126 received placebo. In the exposure–response model for the time course of tumor size, lenvatinib exposure has a saturable effect (*E*_max_) on tumor inhibition with resistance. Tumor growth rate was described in the model with a first-order growth rate constant (i.e., an *E*_max_ model with first-order rate constant [*K*_G_]). The model is described by the following differential equation:

$$\begin{gathered} \frac{{{\text{d}}y(t)}}{{{\text{d}}t}}={K_{\text{G}}}~ \cdot y(t) - \frac{{{E_{\hbox{max} }} \cdot {\text{AVAUC}}}}{{{\text{AVAUC}}+{\text{E}}{{\text{C}}_{50}} \cdot R(t)}} \cdot ~y(t), \hfill \\ R(t)=\exp (\lambda \cdot t), \hfill \\ \end{gathered}$$

where *y* is the sum of diameters of all target lesions (measured per Response Evaluation Criteria in Solid Tumors version 1.1 [14]), *K*_G_ is the first-order tumor growth-rate constant (1/week), *E*_max_ is the maximum change in tumor size in week^−1^ (1/week), EC_50_ is the exposure to lenvatinib based on the average dose between two tumor assessments [average area under the curve (AVAUC) in µg·h/mL] that will achieve 50% of *E*_max_, λ is the parameter for resistance term in week^−1^, *R*(*t*) is a resistance function that incorporates a rate constant of resistance appearance [λ (per week)], which increases EC_50_ with time, and AVAUC is the exposure of lenvatinib based on average dose between two tumor assessments. The effects of interindividual variability (IIV) on *K*_G_ and *E*_max_ and λ were described by an additive error model. IIV on EC_50_ was described by an exponential error model. Residual error was described by a combined additive and proportional error model.

No covariate analyses were performed. Estimation of model parameters was performed using the first-order conditional estimation method with interaction (FOCEI). Goodness-of-fit plots and visual predictive checks were used to evaluate the exposure–response model for tumor-growth inhibition.

### Exposure–response model for safety: dose-altering AEs

The AE data for this model came from 7914 weekly AE reports performed for 24 weeks from patients with RR-DTC in SELECT. Although 392 patients participated in the SELECT trial, 1 patient in the lenvatinib arm did not have PK data. Therefore, the final dataset included 391 patients: 260 patients in the lenvatinib arm and 131 patients in the placebo arm.

Details on the assessment and recording of AE data during the trial have been previously reported [5]. A dose-altering AE was defined as any treatment-related AE leading to study-drug dose interruption, dose reduction, or drug withdrawal. Based on these data, a proportional odds model was developed for the probability of experiencing any dose-altering AE by assigning one event per week to each patient and relating this to lenvatinib AUC based on the highest dose during the week.

Dose-altering AEs were then graded for use in a cumulative logit model for ordinal categorical data: grade 1 was any treatment-related AE leading to dose interruption, and grade 2 was any treatment-related AE leading to dose reduction or study drug withdrawal. Logits of these probabilities (*A*_*i*_) were presented as an effect of lenvatinib (EFF), the baseline values of a particular grade or lower (*B*_*i*_), and subject-specific random effect (η) determining the individual sensitivity assumed to be normally distributed with variance ω^2^ and mean 0. The lenvatinib-exposure parameter tested was lenvatinib AUC based on the highest dose during the week of the event. The effect of lenvatinib exposure was described as an *E*_max_ function:

$${\text{EFF}}~=~\frac{{{E_{\hbox{max} }}\cdot{\text{AUC}}}}{{{\text{E}}{{\text{C}}_{50}}+{\text{AUC}}}}+\eta ;$$

$${A_1}~={B_1}+{\text{EFF}}$$

$${A_2}~={B_2}+{\text{EFF}}$$

$${C_i}~=\exp ({A_i}),\quad ~i~=1,2;$$

$$P\left( {{\text{AE}}~ \ge i} \right)=\frac{{Ci}}{{1+Ci}},\quad i=1,2.$$

Using the final cumulative logit model, the probability of a particular grade of dose-altering AE was then described using the following equations:

$$P({\text{AE}}=2)=~P({\text{AE}} \ge 2);$$

$$P({\text{AE}}=1)=~P({\text{AE}} \ge 1) - P({\text{AE}} \ge 2);$$

$$P({\text{AE}}=0)=1~ - ~P({\text{AE}} \ge 1),$$

where *P*(AE = 2) is probability of having an AE leading to dose reduction/study drug withdrawal, *P*(AE = 1) is probability of having an AE leading to dose interruption, and *P*(AE = 0) is probability of having no dose-altering AE.

No covariate analyses were performed. The marginal likelihood was approximated by using the Laplacian method in nonlinear mixed-effect modeling (NONMEM). Five hundred datasets with identical design to the original dataset were simulated using the parameters from the final model, and the time course of probability was calculated from the simulated data and compared to observed data.

### Dosing-regimen simulations

Lenvima^®^ is formulated as a 4 mg or 10 mg hypromellose hard capsule. Therefore, the efficacy and safety exposure–response models were used to simulate the outcome for the following seven dosing regimens: lenvatinib 24 mg without dose up-titration (the approved reference regimen), lenvatinib 20 mg without dose up-titration, lenvatinib 18 mg without dose up-titration, lenvatinib 14 mg without dose up-titration, lenvatinib 20 mg with dose up-titration, lenvatinib 18 mg with dose up-titration, and lenvatinib 14 mg with dose up-titration. For dose regimens allowing up-titration, the maximum dose was 24 mg, with an up-titration interval of 4 weeks. The dose-adjustment strategies are provided in detail in Online Resource Table 1 (for dose regimens without up-titration) and Online Resource Table 2 (for dose regimens with up-titration). For the 14 mg, 18 mg, and 20 mg doses, the dose will be up-titrated if no dose reduction occurred during the prior 4 weeks. If no dose reduction occurred during the following 4 weeks, the dose could be further up-titrated.

The safety exposure–response model and individual sensitivity (η) values were used to simulate dosing history for six cycles (24 weeks). The average lenvatinib dose across 24 weeks and the proportion of patients with at least one dose reduction due to an AE were computed at replication level. For each of the 7 regimens, 500 replicates for 260 lenvatinib recipients were simulated.

Using the simulated dosing histories, the exposure–response model was applied to predict the time course of tumor-size change over 24 weeks. The simulated tumor size profiles were then used to derive the ORR at 24 weeks. Tumor assessments were performed every 8 weeks, and objective response was defined as a decrease of at least 30% below baseline tumor size. All comparisons between dosing regimens based on model simulations were descriptive.

### Statistical software

Exposure–response analyses for tumor size and dose-altering AEs were performed using NONMEM version 7.2.0 (ICON Development Solutions, Ellicott City, MD, USA) interfaced with PDxPop 5.0 (ICON Development Solutions, Ellicott City, MD, USA). Simulations were performed using SAS software (Version 9.3; SAS Institute, Cary, NC, USA) for dose-altering AEs and NONMEM for tumor size. R software (version 2.10.1; R Foundation for Statistical Computing, Vienna, Austria) was used to generate all plots.

## Results

### Exposure–response model for time course of tumor size

**Fig. 1:**
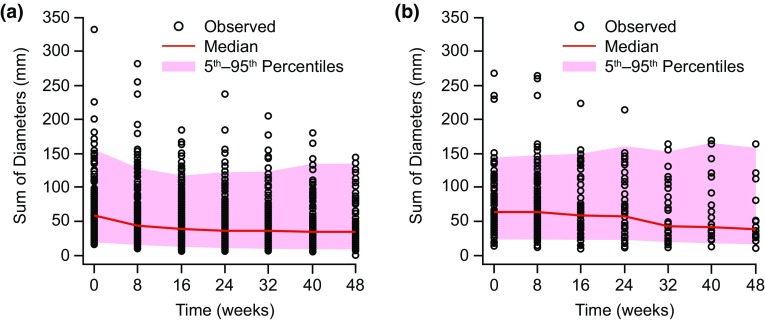
Visual predictive check of observed and model-predicted tumor size with lenvatinib (**a**) and placebo (**b**) in an exposure–response model for time course of tumor size

The parameter estimates for this model using an *E*_max_ function and with tumor growth modeled as first-order *K*_G_ are presented in Table 1. The visual predictive check of the model is shown in Fig. 1, comparing simulated tumor-size data with observed tumor-size data for lenvatinib (Fig. 1a) and placebo (Fig. 1b) (number of replications = 500). The visual predictive check showed good prediction of the data by the model, with more than 90% of the observed tumor-size data falling within the 90% PI.

**Table 1:** Parameter estimates for exposure–response model for time course of tumor size

Parameter (units)	Point estimate	% RSE
*K*_G_ (/week)	0.000932	63.0
*E*_max_ (/week)	0.0834	16.2
EC_50_ (µg h/mL)	2.03	32.1
λ (/week)	0.170	8.47
Interindividual variability		
*K*_G_ IIV SD (/week)	0.00858	6.60
*E*_max_ IIV SD (/week)	0.0508	31.3
EC_50_ IIV (CV %)	40.5	77.4
λIIV SD (/week)	0.0791	28.1
Residual variability		
Proportional (CV %)	3.89	4.79
Additive SD (mm)	1.74	3.18

*CV* coefficient of variation, *E*_*max*_ maximum effect of lenvatinib on tumor suppression, *EC*_*50*_ lenvatinib average area under the concentration–time curve that results in 50% of *E*_max_, *IIV* interindividual variability, *K*_*G*_ tumor growth rate, *SD* standard deviation; *% RSE* percent relative standard error of the estimate = SE/parameter estimate × 100, *λ* resistance term

The time course for tumor size is considered to be well defined by the exposure–response model based on the precision of parameter estimates and predictive checks, as well as goodness-of-fit plots (data not shown) and the individual observed and model-predicted time course of tumor-size plots (data not shown).

### Exposure–response model for dose-altering AEs

**Fig. 2:**
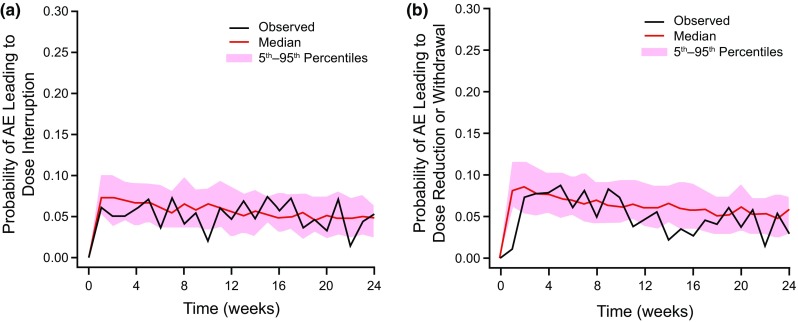
Observed and simulated probabilities of dose-altering adverse events as a predictive check of the exposure–response safety model: adverse events leading to dose interruption (**a**), and adverse events leading to dose reduction or withdrawal of study drug (**b**). *AE* adverse events

Table 2 shows the parameter estimates for the exposure–response model for dose-altering AEs. The predictive check between simulated and observed data (Fig. 2) generally agreed.

**Table 2:** Parameter estimates derived from the proportional odds model for the probability of experiencing any AE leading to dose interruption or dose reduction/drug withdrawal in patients with RR-DTC taking either lenvatinib or placebo

Parameter (units)	Point estimate	% RSE
B1	− 5.64	5.14
B2–B1	− 0.830	6.07
*E*_max_	6.55	11.4
EC_50_ (µg h/mL)	2.95	26.3
IIV (SD)	1.06	18.4

*B1* baseline odds for experiencing an adverse event leading to dose interruption, *B2–B1* baseline odds for experiencing an adverse event leading to dose reduction/withdrawal, *E*_*max*_ maximum effect of lenvatinib, *EC*_*50*_ lenvatinib area under the concentration–time curve that results in 50% of *E*_max_, *IIV* interindividual variability, *RR-DTC* radioiodine-refractory differentiated thyroid cancer, *SD* standard deviation, *%RSE* percent relative standard error of the estimate = SE/parameter estimate × 100

### Simulating the efficacy and safety of different dosing regimens

Simulated average dose and proportion of patients with at least 1 dose reduction during 24 weeks of treatment for each of the seven dosing regimens assessed are shown in Table 3. The currently approved starting dose of lenvatinib is 24 mg once daily. This dosage in the simulation using the exposure–response model for dose-altering AEs was predicted to result in 68.5% of patients during 24 weeks to have at least one dose reduction. The proportion of patients with at least 1 dose reduction was lower (46.9–60.8%) in the regimens with lower starting doses of lenvatinib (20, 18, and 14 mg without up-titration) (Table 3). In contrast, the lower starting doses of lenvatinib of 14, 18, and 20 mg in the dose regimens with up-titration appeared to have similar or slightly lower incidence of dose reductions compared to the approved 24 mg starting dose.

**Table 3:** Simulated average dose of lenvatinib and proportion of patients with ≥ 1 dose reduction due to an adverse event during 24 weeks of treatment

Lenvatinib dosing regimen (mg)	Average dose (mg/day)	Patients with dose reductions (%)			
		≥ 1	≥ 2	≥ 3	≥ 4
Without up-titration					
24	19.23	68.5	33.8	11.2	2.7
20	16.09	60.8	22.3	5.4	1.2
18	15.13	57.3	20.8	5.4	1.2
14	12.15	46.9	12.3	2.7	0.4
With up-titration					
20	18.35	67.7	30.4	8.1	1.9
18	17.90	67.7	28.1	7.7	1.9
14	16.90	65.4	23.8	5.0	0.8

**Fig. 3:**
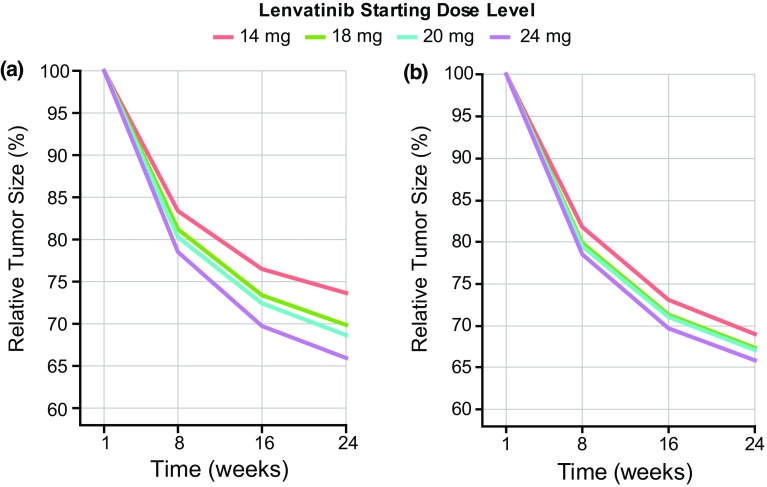
Simulated change from baseline in tumor size over 24 weeks for lenvatinib dosing regimens without up-titration (**a**) and with up-titration (**b**), and at 24 mg. Simulations were generated using the exposure–response model for time course in tumor size

Simulations comparing tumor response over 24 weeks of the seven different dosing regimens showed that the currently approved dosing regimen of lenvatinib 24 mg had larger and earlier reductions in tumor size compared with all the lower starting doses without up-titration (Fig. 3a). Reductions in tumor size with the lower starting doses of lenvatinib were greater when up-titration was allowed but were not as large, or observed as early in the time course of treatment, when compared with the approved 24 mg dose (Fig. 3b).

For lenvatinib 24 mg, the derived ORR at 24 weeks, using the simulated tumor-size profiles, was 50.0%, which was the highest of all the simulated dosing regimens. The ORRs for the lower lenvatinib starting doses of 20, 18, and 14 mg (without up-titration) were lower: 43.5%, 41.5%, and 30.2%, respectively. The ORR with lenvatinib 14 mg was considered to be clinically significantly lower than that for the other doses and therefore was not expected to yield comparable efficacy to the 24 mg dose. The ORRs for lenvatinib 20, 18, and 14 mg starting doses with up-titration to 24 mg were similar or better than the lower doses without up-titration (46.8%, 46.0%, and 41.7%, respectively). However, the reduction in tumor size with the approved lenvatinib 24 mg dose was still larger, and the tumor-size reduction occurred earlier than simulated with any of the lower starting doses, with or without up-titration.

## Discussion

In this study, we developed exposure–response models for tumor-growth inhibition and for dose-altering AEs, and used them to simulate the potential efficacy and safety of seven lenvatinib dosing regimens, including the approved 24 mg dose, in patients with RR-DTC. Longitudinal tumor data of the sum of the target lesion diameter by independent review from both the lenvatinib and placebo arms were used. The thyroid tumor natural *K*_G_ was estimated to be 0.000932 per week. The value of *K*_G_ was comparable to the values reported in the literature [15]. Results of simulations run using the two models showed good agreement with observed data from SELECT for lenvatinib 24 mg in patients with RR-DTC, supporting the suitability of these exposure–response models for simulation of lenvatinib toxicity-related dose modifications.

The model for dose-altering AEs predicted that during 24 weeks of treatment, 68.5% of patients would experience at least 1 dose reduction with the approved lenvatinib 24 mg dose; in comparison, 67.8% of all patients in SELECT had dose reductions (over the entire treatment phase prior to data cutoff) (Eisai data on file) [5]. Similarly, simulations predicted an ORR of 50.0% at 24 weeks for the lenvatinib 24 mg dose, which was in close agreement with results from SELECT (57.5% at 24 weeks) (Eisai data on file).

These simulations predict a more favorable safety profile with the lower doses of lenvatinib 14 and 20 mg without up-titration because the proportions of patients with at least one dose reduction during 24 weeks were 46.9% and 60.8%, respectively, vs. 68.5% with the 24 mg dose. However, ORR (at 24 weeks) was predicted to be lower with lenvatinib 14 mg compared with the approved lenvatinib starting dose of 24 mg. Based on the simulated ORR, the lenvatinib dose regimens allowing up-titration to 24 mg were predicted to have better efficacy compared with the regimens without up-titration; however, this advantage was offset by a worsening of the safety profile for the 18 and 20 mg doses. Thus, either a lenvatinib 14 mg dose with up-titration or an 18 mg dose without up-titration were considered as possible regimens to provide comparable efficacy (ORR at 24 weeks) but a better safety profile than the approved 24 mg dose. The lenvatinib 18 mg regimen without up-titration was selected for simplicity of administration and study conduct for Study 211 (NCT02702388).

Our analysis did have several limitations in addition to the inherent inadequacies of simulation modeling. We explored early dose modification as the safety end point, therefore, cumulative or chronic drug toxicity was not considered. In addition, all treatment-emergent AEs (TEAEs) leading to study drug modification were treated as 1 event in this exposure–response analysis; namely, the same exposure–response relationship was assumed for all TEAEs. For the tumor size model development, only target lesions were considered, and neither nontarget lesions nor new lesions were included. An assumption in the simulations of tumor size for the various dosing regimens is that the empirical resistance function (parameter λ) is identical for all dosing regimens. However, this may be inconsistent with biological expectations. Furthermore, although the model demonstrated good predictive performance, it failed to fully capture the wide heterogeneity present in the data. Additional modifications to improve the model in the future (e.g., including covariate analyses) are warranted.

The average lenvatinib dose from the simulation of 24 weeks of treatment was 19.23 mg/day with the 24 mg dose, which is similar to that reported in SELECT (mean dose of 17.2 mg/day) [5]. This supports the simulation scheme for lenvatinib dosing chosen in this study. Previous PK/pharmacodynamic modeling studies have been used to examine dosing regimens of other multitargeted kinase inhibitors, lending further support to our approach [15, 16]. Of note, although the efficacy end point for this exposure–response analysis was ORR rather than PFS or overall survival, tumor size modeling serves as a treatment efficacy metric that relatively closely reflects survival [17], and modeling the time course of tumor size is a recognized tool in pharmacometric research [13, 17].

Because these simulation data indicated that it is unlikely that the lenvatinib 20 mg dosing regimen would provide sufficiently different exposure and that the 14 mg dose without up-titration was likely to result in a much lower ORR compared with the 24 mg dose, a decision was made that enrollment into Study 211 [12] should be temporarily halted. The lenvatinib 20 mg and 14 mg doses without up-titration were removed from the study and the clinical protocol was revised to include the lenvatinib 24 mg dose without dose escalation and at least 1 alternative lower dosing arm. The study is now recruiting patients to be randomized to receive either a lenvatinib 24 mg or 18 mg starting dose without up-titration, which was approved by both the FDA and EMA.

In conclusion, our PK/pharmacodynamic model and follow-up simulations showed that the recommended high relative starting dose of lenvatinib of 24 mg once daily in patients with RR-DTC appears to provide the best efficacy, but lower starting doses may accomplish comparable antitumor efficacy with a similar or better safety profile.

## Electronic supplementary material

Below is the link to the electronic supplementary material.

Supplementary material 1 (DOCX 21 KB)

Supplementary material 2 (DOCX 23 KB)
