# Supplementary material for: Exposure–response analysis and simulation of lenvatinib safety and efficacy in patients with radioiodine-refractory differentiated thyroid cancer
Source: Cancer Chemother Pharmacol. 2018 Sep 22;82(6):971–8. doi: 10.1007/s00280-018-3687-4 (PMC6267706; doi:10.1007/s00280-018-3687-4)
Supplement: Supplementary file 1 — Supplementary material 1 (DOCX 21 KB) [file 280_2018_3687_MOESM1_ESM.docx]

**SUPPLEMENTAL APPENDIX**

**Supplementary Table 1.** Lenvatinib dose-adjustment strategy for dosing regimens without
up-titration used in the drug-exposure safety-model simulation of dosing history.

| **Lenvatinib dose level (mg)** | | | | |
| --- | --- | --- | --- | --- |
| **Starting dose (1^st^)** | **2^nd^** | **3^rd^** | **4^th^** | **Subsequent** |
| 24 | 20 | 14 | 10 | 0 |
| 20 | 14 | 10 | 8 | 0 |
| 18 | 14 | 10 | 8 | 0 |
| 14 | 10 | 8 | 4 | 0 |
