# Supplementary material for: Exposure–response analysis and simulation of lenvatinib safety and efficacy in patients with radioiodine-refractory differentiated thyroid cancer
Source: Cancer Chemother Pharmacol. 2018 Sep 22;82(6):971–8. doi: 10.1007/s00280-018-3687-4 (PMC6267706; doi:10.1007/s00280-018-3687-4)
Supplement: Supplementary file 2 — Supplementary material 2 (DOCX 23 KB) [file 280_2018_3687_MOESM2_ESM.docx]

**SUPPLEMENTAL APPENDIX**

**Supplementary Table 2.** Lenvatinib dose-adjustment strategy for dosing regimens with
up-titration used in the drug-exposure safety-model simulation of dosing history.

| **Starting dose (mg)** | **Up-titration number** | **Lenvatinib dose level (mg)** | | | | |
| --- | --- | --- | --- | --- | --- | --- |
|  |  | **1^st^** | **2^nd^** | **3^rd^** | **4^th^** | **Subsequent** |
| **20** | 0 | 20 | 14 | 10 | 8 | 0 |
|  | 1 | 24 | 20 | 14 | 10 | 0 |
| **18** | 0 | 18 | 14 | 10 | 8 | 0 |
|  | 1 | 24 | 18 | 14 | 10 | 0 |
| **14** | 0 | 14 | 10 | 8 | 4 | 0 |
|  | 1 | 20 | 14 | 10 | 8 | 0 |
|  | 2 | 24 | 20 | 14 | 10 | 0 |
